# Zero-leak prediction during major lung resection aiming for minimal chest drainage duration: a retrospective analysis

**DOI:** 10.1186/s13019-024-02620-2

**Published:** 2024-03-13

**Authors:** Kuniyo Sueyoshi, McAndrew Merlini, Kosuke Otsubo, Fumitsugu Kojima, Toru Bando

**Affiliations:** Department of Thoracic Surgery, St Luke’s International Hospital, Akashi-Cho 9-1, Chuo-ku, Tokyo, 104-8560 Japan

**Keywords:** Air leak episode, Anatomical pulmonary resection, Early chest tube removal

## Abstract

**Background:**

Early chest tube removal should be considered to enhance recovery after surgery. The current study aimed to provide a predictive algorithm for air leak episodes (ALE) and to create a knowledge base for early chest tube removal.

**Methods:**

This retrospective study enrolled patients who underwent thoracoscopic anatomical pulmonary resections in our unit. We defined ALE as any airflow ≥ 10 mL/min recorded in the follow-up charts based on the digital thoracic drainage device. Multivariate regression analysis was used to control for preoperative and intraoperative confounding factors. The ALE prediction algorithm was constructed by combining an additive ALE risk-scoring system using the coefficients of the significant predictive factors with the intraoperative water-sealing test.

**Results:**

In 485 consecutive thoracoscopic major pulmonary resections, ALE developed in 209 (43%) patients. Statistically significant ALE-associated preoperative factors included male sex, lower body mass index, radiologically evident emphysema, lobectomy, and upper lobe surgery. Significant ALE-associated intraoperative factors were incomplete fissure and pleural adhesion. The ALE risk scoring demonstrated an average area under the receiver operating characteristic curve of 0.72 in the fivefold cross-validation test. The ALE prediction algorithm correctly predicted ALE-absent patients at a negative predictive value of 80%.

**Conclusions:**

The algorithm may promote the optimization of the chest tube-dwelling duration by identifying potential ALE-absent patients for accelerated tube removal.

**Supplementary Information:**

The online version contains supplementary material available at 10.1186/s13019-024-02620-2.

## Background

The placement of chest drainage tubes after pulmonary resections has a negative impact on postoperative chest pain and ventilatory function [1]. Surgeons should consider removing the chest tubes as early as possible to encourage early mobilization, reduce opioid analgesic requirements, and prevent potential complications to enhance recovery after surgery [2]. Hence, tube removal on the day of surgery is ideal. A few institutes have reported the safety and benefits of early drain removal procedures in selected patients who underwent anatomical lung resection [3, 4]. However, most surgeons still prefer conservative drainage management and keep the chest tubes in place, at least until the day after the lobectomy or segmentectomy. This tendency is largely due to their concerns regarding potential risks, such as postoperative pulmonary fistula development.

Prolonged air leak (PAL) lasting 5–7 days after surgery is a common complication of pulmonary resections. To date, many studies have described the frequency of PAL occurrence and its associated risk factors, including smoking, limited pulmonary function, comorbidities, lower body mass index (BMI), upper lobe surgery, and the presence of pleural adhesion [5]. Accumulated data associated with PAL has provided evidence to reasonably predict its development [6] with an accuracy of a certain degree [7] and appropriately manage this complication [8, 9].

Meanwhile, little attention has been given to air leak episodes (ALE), defined as any air leak event after lung surgery. Although ALE is not a postoperative complication, we believe that the prediction of ALE development would provide valuable information and help further support the concept of accelerated drain removal. Thus, the present study aimed to provide an ALE prediction algorithm to discern candidates for chest tube removal immediately after surgery. First, we revealed the frequency and patterns of ALE development associated with thoracoscopic lobectomy and segmentectomy. Quantitative air leakage information was obtained from the digital thoracic drainage system to detect latent ALE development. Subsequently, we constructed the ALE prediction algorithm consisting of two evaluation steps: the pre-resection assessment based on the ALE risk-scoring system and the post-resection assessment based on the routine intraoperative water-sealing test.

## Methods

### Study patients

**Fig. 1:**
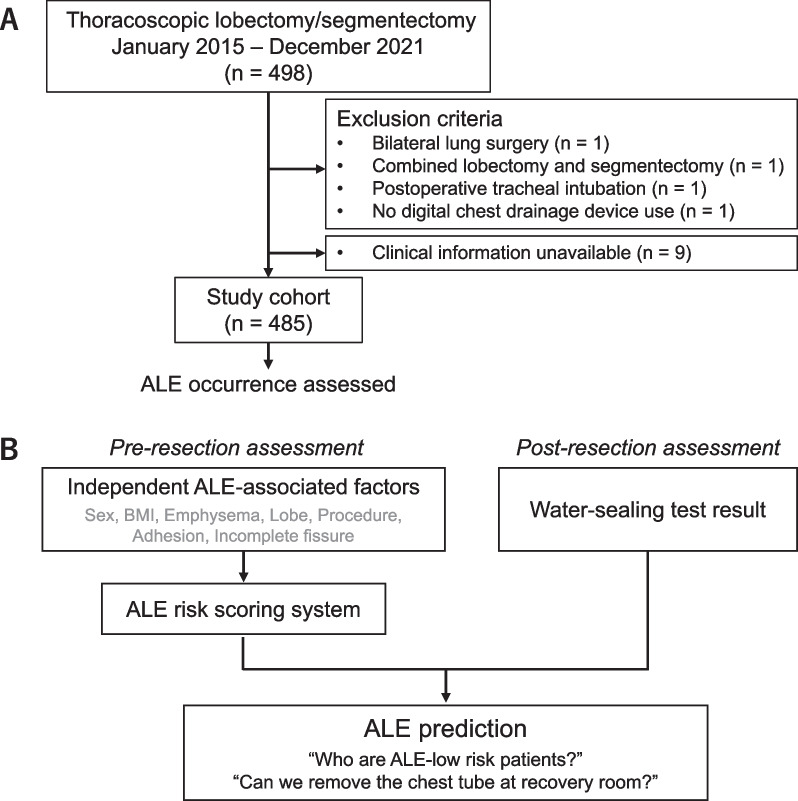
Overview of the study. **A** Patient flow. **B** Outline of ALE prediction

Consecutive patients who underwent thoracoscopic (video-assisted thoracic surgery [VATS] or robot-assisted thoracic surgery [RATS]) anatomical pulmonary resections, including lobectomy and segmentectomy, from January 1, 2015, through December 31, 2021, in our institute were enrolled in this retrospective study (Fig. 1A). Exclusion criteria were bilateral lung surgery (n = 1), extended lung resection beyond the scope of standard lobectomy or segmentectomy (n = 1 for combined lobectomy and segmentectomy), postoperative tracheal intubation management (n = 1), and the use of a conventional 3-bottle chest drainage device (n = 1). Patients with incomplete clinical information were excluded from the following analysis (n = 9). This study was approved by the institutional review board of St. Luke’s International Hospital (21-R177). The requirement for written informed consent was waived, and the opt-out method was used.

### Data collection

Preoperative factors collected from electronic medical records included baseline clinical features, serum albumin concentration, preoperative pulmonary function test results, and the radiological diameter of lung tumors (including ground-glass sections) recorded before the initiation of the study. Additionally, surgical information, including the affected lobe and procedure, and intraoperative findings (e.g., adhesion, incomplete fissure, and intraoperative water-sealing test results) were extracted from the surgical records. In addition, all serial records of volumetric air leak flow, which had been measured by the digital thoracic drainage device (Thopaz®, Medela, Inc., Baar, Switzerland) and documented at each medical check-up timepoint, were gathered to identify ALE occurrence.

### Operative procedures

Thoracoscopic surgery in this study refers to thoracoscopic lung resection performed through a maximum of an 8 cm (typically 4–5 cm) utility incision plus two to four (typically two) smaller accessory ports. Interlobar and intersegmental lines were resected solely using staplers to minimize air leaks. This principle was also applied to the division of lung parenchyma near the hilum in segmentectomy; these procedures did not usually involve electro-sections along intersegmental veins unless electro-sections were required from the technical or oncological viewpoints. Intersegmental lines were identified primarily by the bronchoscopic multi-spot dye-marking technique (Virtual Assisted Lung Mapping [VALMAP] [10]) for invisible lesions.

The water sealing test was manually performed by gradually increasing the intra-tracheal pressure up to 25 cm H_2_O. The test result was positive when continuous air bubbles were detected at any leak point, including the bronchiole fistula, lung parenchyma lacerations, and stapler lines. Fibrin glue mist was sprayed on the dissected lung surface when substantial bubbling was evident during the water submersion test, severe emphysema was present, or surgeons anticipated air leaks.

### Postoperative management

The digital thoracic drainage device (Thopaz®) was used for postoperative chest drainage. The “regulated” Thopaz® suction pressure was typically set to − 8 cm H_2_O, which is considered equivalent to physiological pressure. The chest tubes were removed when the air leak was ≤ 20 mL/min at − 8 cm H_2_O for 8 h. This removal criterion was set according to a previous study [11] and has been adopted throughout the study period in our department. The indication for pleurodesis was continuous, non-declining air leaks lasting more than three days. Blood patch procedures were first applied one to three times. If the air leaks did not cease or decline, we used OK-432. When pleurodesis was ineffective, reoperation was performed. There were no cases where endobronchial embolization was performed.

### Outcomes

The primary outcome was the incidence of ALE. We defined ALE as any airflow ≥ 10 mL/min recorded in the follow-up charts. The details of the chart recording are described below. The secondary outcome was the incidence of PAL, defined as an air leak lasting ≥ 5 days after surgery.

### ALE measurement

Well-trained nurses in the thoracic surgery department recorded flow volume trends observed in 2–5 min periods during routine medical check-ups based on the measurements of the digital thoracic drainage device. Leak volume checks were typically done on arrival at the ward, every 1 h for the first 4 h post-surgery, every 2 h until the morning of postoperative day (POD) 1, and every 6 h after that with patients in a supine position.

### Statistical analysis

Univariate and multivariate logistic regression analyses were used to estimate the odds ratio (OR) of the incidence of ALE. Welch’s t-test, Mann–Whitney U-test, and Fisher’s exact test were performed to assess the null assumption of equality of continuous variables, time variables, and the independence of categorical variables, respectively. Statistical significance was set at an alpha level of 0.05.

The ALE prediction algorithm was composed of two steps: (1) Pre-resection assessment with an ALE scoring system and (2) post-resection assessment with a water-sealing test (Fig. 1B). (1) The ALE scoring was formulated using the coefficients of the significant predictive factors in the multivariate regression analysis. First, continuous variables were discretized at the threshold obtained by approximating the “top left” value of the best ALE-predictive ability in the univariate receiver operating characteristic (ROC) curve plot (e.g., BMI < 22 vs. BMI ≥ 22). Second, the coefficients of the multivariate regression model were rounded to a 0.5-increment number to obtain the corresponding scores. Those scores were summed up to calculate the total ALE risk score; then, a threshold was set to the “top left” value of the best diagnostic ability on the ROC curve. (2) The ALE prediction algorithm was then constructed by integrating the ALE risk-scoring system and water-sealing test results. Analyses were performed using the R statistical software (R Foundation for Statistical Computing, Vienna, Austria. Version 3.6.3). All codes used above are available via https://github.com/Kuniyo-Sueyoshi/PoAL.

## Results

A total of 485 patients (primarily male, 56%) with a mean age of 66 years (standard deviation [SD], 11) were included in the analysis (Fig. 1A, Table 1). Primary lung carcinoma (92%) with a relatively small radiological gross diameter (2.0 cm on average) was the predominant diagnosis in this cohort. The proportion of surgical procedures performed (50% for segmentectomy vs. 50% for lobectomy), and the proportion of the surgical lobe location (58% for the upper lobe vs. 42% for the other lobes), were well balanced. Thirty-three patients (6.8%) required pleurodesis to manage their postoperative pleural fistula (Table 2). PAL (> 5 days) developed in 13 patients (2.7%). The chest drain was removed on the 1.7th POD on average (median 1, IQR 1) across all cases. The mean length of hospital stay was 5.0 days (median 4, IQR 2). The median interval between each leak flow record was 135.0 min (1st and 3rd IQR, [88.7, 196.5]).

**Table 1:** Preoperative and intraoperative factors

Variable	Overall (n = 485)^a^	ALE-absent (n = 276)^a^	ALE-present (n = 209)^a^	*p* value
Age	65.6 (11.0)	64.5 (11.0)	67.0 (10.9)	0.016^b^
Sex				0.003^c^
Male	274 (56%)	140 (51%)	134 (64%)	
Female	211 (44%)	136 (49%)	75 (36%)	
BMI	23.2 (3.7)	23.6 (3.8)	22.5 (3.5)	0.001^b^
Pack-year	24.0 (29.8)	20.9 (27.4)	28.2 (32.2)	0.009^b^
%Predicted FEV_1.0_	73.8 (9.4)	74.0 (8.8)	73.4 (10.1)	0.48^b^
Emphysema present	129 (27%)	50 (18%)	79 (38%)	< 0.001^c^
DM present	63 (13%)	35 (13%)	28 (13%)	0.82^c^
IVD history present	35 (7.2%)	16 (5.8%)	19 (9.1%)	0.17^c^
Albumin [mg/dL]	4.28 (0.34)	4.29 (0.35)	4.25 (0.32)	0.15^b^
Tumor size [cm]	2.0 (1.2)	1.9 (1.1)	2.2 (1.4)	0.017^b^
Diagnosis				0.50^c^
Lung carcinoma	447 (92%)	252 (91%)	195 (93%)	
Metastasis/Benign	38 (7.8%)	24 (8.7%)	14 (6.7%)	
Lobe				0.002^c^
Lower/Middle	203 (42%)	132 (48%)	71 (34%)	
Upper	282 (58%)	144 (52%)	138 (66%)	
Approach				0.31^c^
RATS	39 (8.0%)	19 (6.9%)	20 (9.6%)	
VATS	446 (92%)	257 (93%)	189 (90%)	
Procedure				< 0.001^c^
Segmentectomy	241 (50%)	170 (62%)	71 (34%)	
Lobectomy	244 (50%)	106 (38%)	138 (66%)	
Staff				0.25^c^
Doctor A	250 (52%)	136 (49%)	114 (55%)	
Doctor B	235 (48%)	140 (51%)	95 (45%)	
Year				0.91^c^
2015–2017	193 (40%)	108 (39%)	85 (41%)	
2018–2019	148 (31%)	84 (30%)	64 (31%)	
2019–2021	144 (30%)	84 (30%)	60 (29%)	
Adhesion area				< 0.001^c^
1. 0–20%	451 (93%)	269 (97%)	182 (87%)	
2. 30–50%	24 (4.9%)	6 (2.2%)	18 (8.6%)	
3. 60–100%	10 (2.1%)	1 (0.4%)	9 (4.3%)	
Incomplete fissure				< 0.001^c^
Grade 1	380 (78%)	234 (85%)	146 (70%)	
Grade 2	90 (19%)	37 (13%)	53 (25%)	
Grade 3	13 (2.7%)	5 (1.8%)	8 (3.8%)	
Grade 4	2 (0.4%)	0 (0%)	2 (1.0%)	

^a^Mean (SD); n (%). ^b^Welch Two Sample t-test. ^c^Fisher's exact test. *BMI* body mass index, *FEV*_*1.0*_ forced expiratory volume in the first second of expiration, *Emphysema*, radiologically evident emphysema, including centrilobular, paraseptal, and panlobular emphysema on computed tomography images. *DM* diabetes mellitus, *IVD* ischemic heart disease and intracerebral disease, *Adhesion area* pleural adhesion coverage where sharp dissection with the electrocautery or energy device is required. *Incomplete* *fissure* Craig and Walker criteria of completeness of fissures [26]. Grade 1, complete fissure; grade 2, complete visceral cleft but parenchymal fusion; grade 3, visceral cleft evident for part of the fissure; grade 4, complete fusion of the lobes

**Table 2:** Surgical outcomes and postoperative measurements

Variable	Overall (n = 485)	ALE-absent (n = 276)	ALE-present (n = 209)	*p* value
Water sealing test^a^				< 0.001^d^
Air bubble (–)	284 (59%)	187 (68%)	97 (46%)	
Air bubble (+)	201 (41%)	89 (32%)	112 (54%)	
PGA sheet used^a^	286 (59%)	155 (56%)	131 (63%)	0.15^d^
Fibrin glue used^a^	119 (25%)	45 (16%)	74 (35%)	< 0.001^d^
Fibrin and PGA sheet used^a^	110 (23%)	41 (15%)	69 (33%)	< 0.001^d^
Operation time [min]^c^	192 (68)	182 (64)	205 (73)	< 0.001^f^
Bleeding [mL]^b^	53 (72)	43 (55)	65 (88)	0.001^e^
Chest tube removal [POD]^c^	1 (1)	1 (0)	2 (2)	< 0.001^f^
PAL present^a^	13 (2.7%)	0 (0%)	13 (6.2%)	< 0.001^d^
Pleurodesis required^a^	33 (6.8%)	0 (0%)	33 (16%)	< 0.001^d^
Re-intervention required^a^	2 (0.4%)	0 (0%)	2 (1.0%)	0.19^d^
Discharge [POD]^c^	4 (2)	4 (2)	5 (2)	< 0.001^f^

^a^n (%). ^b^Mean (SD). ^c^Median (IQR). ^d^Fisher’s exact test. ^e^Welch Two Sample t-test. ^f^Mann-Whitney U test. *PGA* polyglycolic acid, *POD* postoperative days, *PAL* prolonged air leak (> 5th POD); Re-intervention, reoperation or chest tube re-insertion required to manage pleural fistula

**Fig. 2:**
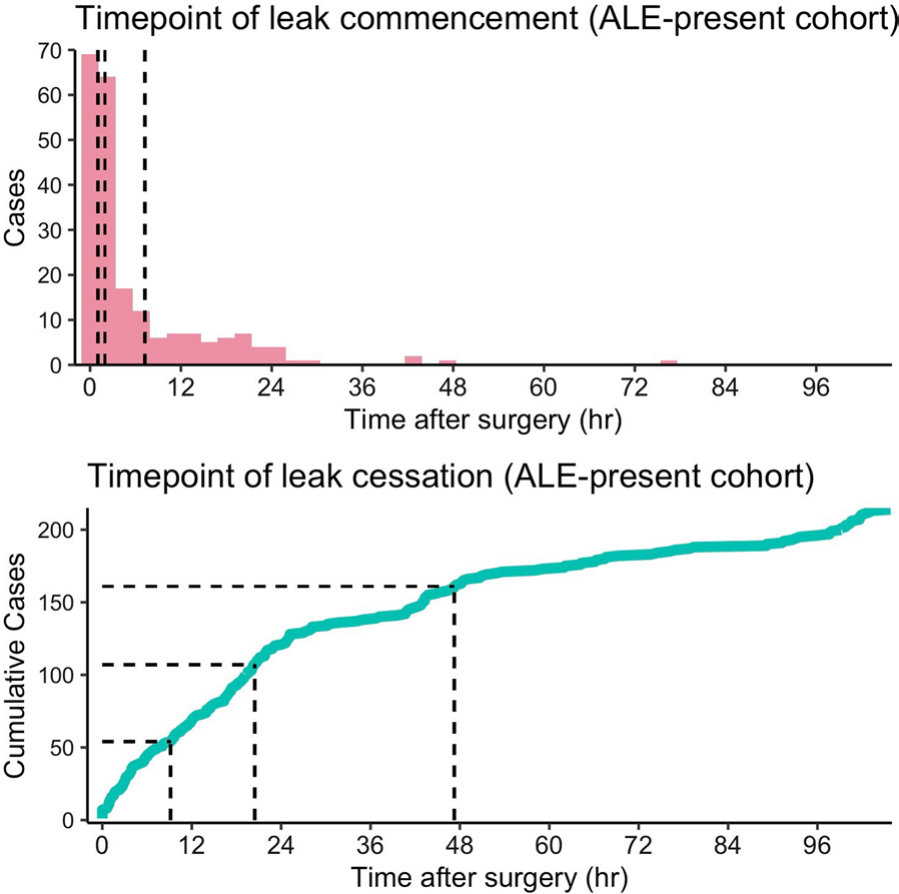
Patterns of ALE development. **A** Timepoints of ALE development documented for the first time after surgery in ALE-present cases. **B** Timepoints of ALE cessation after surgery in ALE-present cases. Vertical dashed lines denote the 1st, 2nd, and 3rd quantile positions of the histograms of cases (**A**) and cumulative curve of cases (**B**)

Two hundred and nine patients (43%) developed ALE at any point after recovery from anesthesia, while the other 276 (57%) demonstrated no air leak up to chest tube removal (Table 1). ALE occurred from the middle of the time course in some cases. For example, more than 25% of the ALE-present patients developed ALE at 6 h or later for the first time (Fig. 2A). Meanwhile, ALE duration varied among patients; 52% of the ALE-present patients had any air leakage after 20 h in the postoperative course. (Fig. 2B). In a univariate analysis, patients who developed ALE were older, more likely to be male, had a lower BMI, a history of tobacco use, emphysema evident on computed tomography findings, and a larger tumor size (Table 1). Patients in the ALE-present group predominantly underwent lobectomy and upper lobe surgery. Intraoperatively, adhesions and incomplete fissures were likely to be observed. No significant differences were noted in the percent predicted forced expiratory volume in one second, diabetes mellitus prevalence, history of intravascular diseases including cardiovascular and cerebral vascular disease, preoperative serum albumin concentration, surgeons, and surgery year-trends between the two groups. Multivariate logistic regression analysis demonstrated that significant preoperative factors associated with the development of ALE were male sex (OR 2.2, CI 1.3–3.6), lower BMI (OR 1.2, CI 1.1–1.3), radiologically evident emphysema (OR 2.0, CI 1.1–3.6), lobectomy (OR 2.9, CI 1.8–4.6), and upper lobe surgery (OR 1.7, CI 1.1–2.6) (Table 3). Significant intraoperative factors associated with ALE development were adhesion (OR 2.9, CI 1.4–6.7) and incomplete fissure (OR 1.9, CI 1.3–3.0).

**Table 3:** Multivariate analysis of preoperative and intraoperative factors

Characteristic	OR^a^	[95% CI^a^]	*p* value
Age	1.01	[0.99, 1.03]	0.18
**Sex:* Male***	2.16	[1.30, 3.63]	**0.003**
**BMI**	0.85	[0.79, 0.91]	**< 0.001**
Pack-year	0.99	[0.98, 1.00]	0.17
**Emphysema:** ***present***	2.00	[1.12, 3.61]	**0.020**
Tumor size	0.97	[0.81, 1.16]	0.70
**Lobe:** ***Upper***	1.72	[1.13, 2.63]	**0.012**
**Procedure:** ***Lobectomy***	2.88	[1.84, 4.56]	**< 0.001**
**Adhesion area**	2.85	[1.40, 6.70]	**0.008**
**Incomplete fissure**	1.92	[1.25, 3.02]	**0.004**

^a^*OR* odds ratio, *CI* confidence interval. Significant factors with p-values less than 0.05 are represented in bold. The factors, adhesion area and incomplete fissure, were implemented as numerical variables ranging 1–3 and 1–4, respectively. See Table 1 for details

To explore the negative association of segmentectomy with ALE development (Table 3), which was somewhat counterintuitive, we further stratified the cohort by the results of the post-resection water sealing test (Additional file 1: Fig. S1A). The leak cessation rate, defined as the proportion of ALE-absent cases in the water-bubble test-positive subgroup, was significantly higher after segmentectomy than after lobectomy. Likewise, the leak commencement rate, the proportion of ALE-present cases in the water-bubble test-negative subgroup, was significantly higher after lobectomy (Additional file 1: Fig. S1B). Polyglycolic acid sheets were predominantly used in segmentectomy (*p* < 0.05), but there was no significant imbalance of surgeons, year trend, or fibrin glue between the two procedures.

**Fig. 3:**
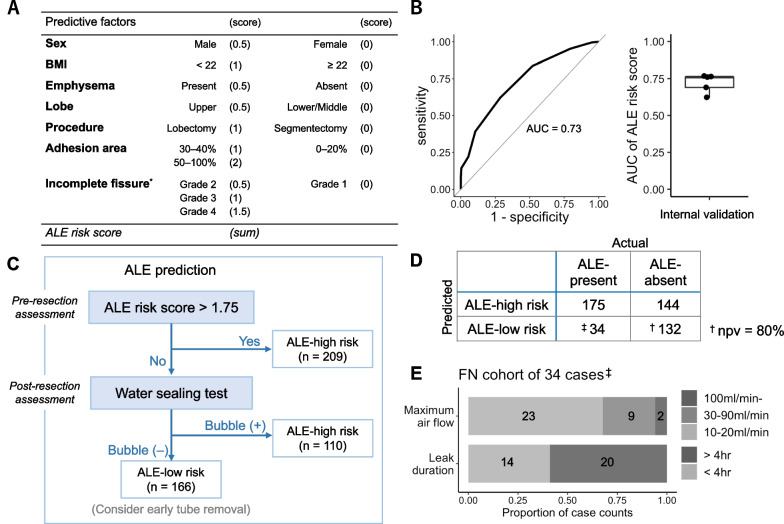
ALE prediction. **A** A representative ALE scoring model developed in a fivefold cross-validation method. **B** ROC curve of the ALE risk scoring system (left) and a box plot showing its performance in fivefold cross-validation (right). **C** ALE prediction algorithm and patient flow. **D** The confusion matrix of the ALE prediction algorithm illustrated in (**C**). A dagger † denotes the true negative cohort, while double daggers ‡ denotes the false negative cohort. **E** Maximum leak flow (top) and leak duration time (bottom) in the false negative (FN) cohort^†^. AUC, area under the curve. ALE risk score, the total score of the ALE scoring model (A). npv, negative predictive value

To construct an easy-to-use algorithm for ALE prediction, we first calculated an ALE risk score based on the coefficients of the multivariate regression model (Fig. 3A). The ALE risk scoring system exhibited a mean area under the curve of 0.72 (SD 0.06) in a five-fold cross-validation test, which was stable concerning the random partitioning procedures into training and test sub-cohorts (Fig. 3B). Subsequently, we assembled the representative scoring system and water-sealing test results to construct an ALE prediction algorithm (Fig. 3C). The algorithm categorized 166 patients into the ALE-low-risk group, a candidate cohort for early drain removal. Of them, 132 patients were actual ALE-absent cases (a negative predictive value of 80%, Fig. 3D) with a mean drain removal of 1.1 days (Median 1, IQR 0) (Table 4). The remaining 34 patients, incorrectly categorized into the ALE-low-risk group, did not develop PAL nor require pleurodesis procedures (Table 4). In addition, the maximum leak flow rate was 10–20 mL/min in 67% and ALE ceased within 4 h in nearly 40% of this false-negative group (Fig. 3E).

**Table 4:** Details of 166 patients predicted as ALE-low-risk group by the algorithm

Predicted	ALE-low-risk cases	
Actual	ALE-absent (n = 132)^a^	ALE-present (n =34)^a^
Pleurodesis		
Not required	132 (100%)	34 (100%)
Required	0 (0%)	0 (0%)
PAL		
Absent	132 (100%)	34 (100%)
Present	0 (0%)	0 (0%)
Re-intervention required		
Not required	132 (100%)	34 (100%)
Required	0 (0%)	0 (0%)
Chest tube removal [POD]	1 (0)	1 (1)
Discharge [POD]	4 (2)	4 (2)

^a^Median (IQR); n (%)

## Discussion

Literature has suggested a positive impact of early chest drain removal on patient experience, analgesic requirement and use, and postoperative physical function [1, 12]. Hence, there is an increasing interest in minimal drainage management to enhance recovery after surgery [13, 14]. A few institutes follow workflows to remove chest tubes on the day of lung surgery by repeatedly checking whether any pleural fistula exits during or after surgery [3, 4]. However, air leaks can occur in varied patterns: intermittent, variable [15], and delayed (Fig. 1). This uncertainty hampers, at least in part, surgeons’ pursuit of accelerated chest tube removal.

In the present study, we integrated a pre-resection scoring system and water-sealing test results to enhance the prediction value. The algorithm correctly predicted 132 patients as ALE-absent with a true negative rate of 80%, who could have their chest tubes removed immediately after surgery from the viewpoint of air leak management. Here, we adopted the stringent criteria of ALE, any airflow ≥ 10 mL/min, to minimize the potential risks of thoracic tube re-insertion in case the algorithm incorrectly predicted ALE-absent patients. A clinically valid threshold for chest tube removal is reported to be more pliable, ranging from 0 to 50 mL/min over 4–8 h [16]. In this regard, it would be notable that the maximum leak flow rate was 10–20 mL/min in 67% and that ALE ceased within 4 h in nearly 40% of our 34 false negative cases (Fig. 3E), suggesting the margin of safety in our algorithm is practically larger than the false negative rate of 20%. Therefore, it may be safe and reasonable to withdraw the chest tube on the day, particularly for patients who undergo surgery in the morning, if they meet the criteria. However, the validity of the algorithm should be evaluated in prospective studies in the future.

There is substantial discordance between water-sealing test results and ALE occurrence (Table 2). This discrepancy may be partly attributed to cough-associated air leaks that start immediately after coughing at extubation, leading to pleural laceration caused by rapidly increased intratracheal pressure. The cough-related ALE may be suppressed by removing trigger stimuli, such as gentle recovery from anesthesia or the use of supraglottic airway devices at extubation [17]. Meanwhile, approximately 25% of air leaks started 6 h or more after surgery (Fig. 1). This cohort needs to be correctly predicted as potential air leak-positive cases. It is also important not to miss air bubbles during the water-submersion test, as inflamed lungs narrow the thoracoscope view, leading to inadequate examination for ALE.

Several ALE-associated factors, including male sex, lower BMI, emphysema, and upper lobe surgery, were noted in this study (Table 3). Those are well-known PAL-related risk factors reported in previous studies [18–20], in line with the ALE’s definition that includes PAL. Interestingly, segmentectomy was one of the negative predictive factors for ALE events (Table 3). The higher ALE cessation rate and lower ALE commencement rate (Additional file 1: Fig. S1B) also support the robustness of the predictive value. This is somewhat counterintuitive, considering that more lung parenchyma resection is needed in procedures involving a segmentectomy rather than a lobectomy. However, varied outcomes have been reported regarding PAL incidence associated with those procedures in propensity-matching studies [21, 22] and safety analysis of a randomized control study [23]. A possible advantage of segmentectomy, if any, is the remnant lung volume in the thoracic cavity. The remaining lobes could be placed against the remaining segments, which may patch over minor pleural lacerations or fix the lung position, preventing weak points of the pleura from being torn by tension, as discussed by Berg et al. [22]. This “remnant lung” hypothesis is concordant with the lower leak incidence in the right middle lobectomy than other lobe lobectomies [24, 25], in the points of the longer parenchymal resection lines, larger volume of remaining lungs, and relative position of the resected lung. In addition, our procedure to use staplers for intersegmental line divisions instead of electrocautery needs to be considered to discuss the surgical procedure factor (see the “Method” section). However, it should be noted that the causality between surgical procedures and ALE was not tested in this study.

There were several limitations to our study design. First, the volume of air leaks was not recorded continuously but intermittently during medical check-ups. Also, inter-observer variations in the airflow records exist due to the different observation durations. A thorough ALE assessment can be conducted using continuous leak data digitally recorded in Thopaz®’s memory. Second, selection bias, owing to the retrospective nature, should be considered. We incorporated the major confounders into the regression models, but we acknowledge that some covariates, such as surgeon's preference, probably remain unadjusted. For example, we omitted the fibrin glue factor from our model because unassessed co-founders, such as surgeons' anticipation of air leaks, probably overwhelmed the fibrin’s ability as sealants, leading to the odds ratio over one (Table 2). Third, drained fluid volume was not assessed, which could impede chest tube removal. Finally, the validation of our ALE prediction algorithm was not sufficient. Although the scoring system exhibited stability in the five-fold cross-validation test (internal validation), we need to plan prospective studies to put the concept of accelerated tube removal into practice.

## Conclusions

We investigated the development of ALE. Our ALE prediction algorithm performed well, although it is yet to be verified using prospectively accumulated data. Accumulating quantitative data of ALE would promote the construction of theoretical frameworks to help surgeons select cases suitable for early tube removal.

### Supplementary Information

**Additional file 1. Supplementary Figure 1**. Leak conversion rate following segmentectomy and lobectomy. **A** Air bubble findings in intraoperative water sealing test and following ALE states. The color bands width represents case numbers. **B** Leak cessation rate represents the proportion of ALE-absent cases in the water-sealing test-positive cohort. Leak commencement rate represents the proportion of ALE-present cases in the water-sealing test-negative cohort. Fisher's exact test was applied.

## Data Availability

The data underlying this article will be shared on reasonable request to the corresponding author with appropriate anonymization. All codes used for analysis are available via https://github.com/Kuniyo-Sueyoshi/PoAL.

## Abbreviations

ALE: Air leak episode
PAL: Prolonged air leak
ERAS: Enhanced recovery after surgery
VATS: Video-assisted thoracic surgery
RATS: Robot-assisted thoracic surgery
ROC: Receiver operating characteristic
AUC: Area under the ROC curve
POD: Postoperative day
FEV_1.0_: The percent predicted forced expiratory volume in one second
BMI: Body mass index
DM: Diabetes mellitus
IVD: Intravascular diseases
