# Supplementary material for: Zero-leak prediction during major lung resection aiming for minimal chest drainage duration: a retrospective analysis
Source: J Cardiothorac Surg. 2024 Mar 13;19:120. doi: 10.1186/s13019-024-02620-2 (PMC10935967; doi:10.1186/s13019-024-02620-2)
Supplement: Supplementary file 1 — Additional file 1. Supplementary Figure 1. Leak conversion rate following segmentectomy and lobectomy. A Air bubble findings in intraoperative water sealing test and following ALE states. The color bands width represents case numbers. B Leak cessation rate represents the proportion of ALE-absent cases in the water-sealing test-positive cohort. Leak commencement rate represents the proportion of ALE-present cases in the water-sealing test-negative cohort. Fisher's exact test was applied. [file 13019_2024_2620_MOESM1_ESM.pdf]

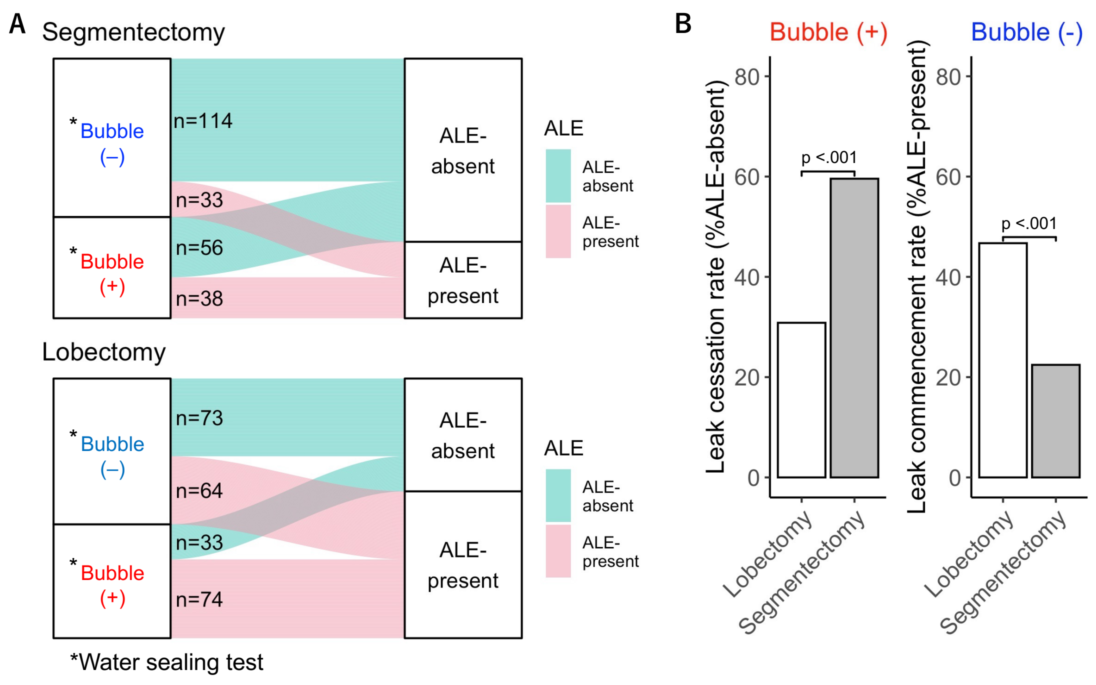


**Supplementary Figure 1.** Leak conversion rate following segmentectomy and lobectomy. (**A**) Air bubble findings in intraoperative water sealing test and following ALE states. The color bands width represents case numbers. (**B**) Leak cessation rate represents the proportion of ALE-absent cases in the water-sealing test-positive cohort. Leak commencement rate represents the proportion of ALE-present cases in the water-sealing test-negative cohort. Fisher's exact test was applied.
